# Impact of the Expression System on Recombinant Protein Production in *Escherichia coli* BL21

**DOI:** 10.3389/fmicb.2021.682001

**Published:** 2021-06-21

**Authors:** Gema Lozano Terol, Julia Gallego-Jara, Rosa Alba Sola Martínez, Adrián Martínez Vivancos, Manuel Cánovas Díaz, Teresa de Diego Puente

**Affiliations:** Department of Biochemistry and Molecular Biology and Immunology (B), Faculty of Chemistry, University of Murcia, Campus of Espinardo, Regional Campus of International Excellence “Campus Mare Nostrum”, Murcia, Spain

**Keywords:** *Escherichia coli*, recombinant protein, expression system, promoter, origin of replication, microbial factory

## Abstract

Recombinant protein production for medical, academic, or industrial applications is essential for our current life. Recombinant proteins are obtained mainly through microbial fermentation, with *Escherichia coli* being the host most used. In spite of that, some problems are associated with the production of recombinant proteins in *E. coli*, such as the formation of inclusion bodies, the metabolic burden, or the inefficient translocation/transport system of expressed proteins. Optimizing transcription of heterologous genes is essential to avoid these drawbacks and develop competitive biotechnological processes. Here, expression of YFP reporter protein is evaluated under the control of four promoters of different strength (P_*T7*_*_*lac*_*, P_trc_, P_tac_, and P_BAD_) and two different replication origins (high copy number pMB1′ and low copy number p15A). In addition, the study has been carried out with the *E. coli* BL21 wt and the *ackA* mutant strain growing in a rich medium with glucose or glycerol as carbon sources. Results showed that metabolic burden associated with transcription and translation of foreign genes involves a decrease in recombinant protein expression. It is necessary to find a balance between plasmid copy number and promoter strength to maximize soluble recombinant protein expression. The results obtained represent an important advance on the most suitable expression system to improve both the quantity and quality of recombinant proteins in bioproduction engineering.

## Introduction

Expression of heterologous and autologous genes is a routine method employed in several biotechnological fields such as metabolic engineering, *in vivo* biocatalysis, or in recombinant proteins or other high-value metabolite production (Sanchez-Garcia et al., 2016; Badenhorst and Bornscheuer, 2018; McCarty and Ledesma-Amaro, 2019). Thus, the strategy followed in a biotechnological process usually includes the expression of enzymes or complete biosynthetic pathways necessary to achieve the compound of interest (Assenberg et al., 2013; Gallego Jara et al., 2015). *E. coli* has traditionally been, and still is, the most used host microorganism in biotechnology. *E. coli* has several advantages to be employed to express heterologous proteins such as fast growth, variety of culture broths, or high number of biology tools designed to obtain genetical modifications (Huang et al., 2012; Blount, 2015; Vargas-Maya and Franco, 2017; Chandran Sathesh-Prabu and Lee, 2018; Xu et al., 2020).

The expression of recombinant proteins is influenced by both physical and transcriptional conditions, such as temperature, shaking, promoter strength, or number of copies of the expression vector used (Aristidou et al., 1999; Balzer et al., 2013; Niu et al., 2014; Bernal et al., 2016; Lozano Terol et al., 2019; Wu et al., 2019). In spite of the advantages, some problems are also associated with the production of recombinant proteins in *E. coli*: the formation of inclusion bodies, the inefficient protein translocation, or the metabolic burden (Mairhofer et al., 2013; Baig et al., 2014; Marschall et al., 2017). Some current strategies developed to solve these drawbacks are focused on supplementation of essential precursors or use of co-cultures (Gurramkonda et al., 2018; Slouka et al., 2019; Chiang et al., 2020; Kumar et al., 2020; Wang et al., 2020). Metabolic burden relates to imbalance suffered by a host cell when a heterologous gene is expressed. The metabolic burden is associated with energetic and precursor constraints due to the transcription and translation of non-essential proteins for the host cell. This limitation is reflected in the alteration of physiological parameters, such as growth rate, and in the downregulation of several essential metabolic pathways for the cell (Mairhofer et al., 2013; Tan et al., 2020; Li and Rinas, 2021). In order to minimize metabolic imbalance, several commercially available plasmids have been engineered to design each biotechnological process, choosing between different promoters and origins of replication, both responsible for the expression level of the gene/s of interest (Zaslaver et al., 2006; De Mey et al., 2007; Wang et al., 2009; Rosano and Ceccarelli, 2014; Yang et al., 2016; Jervis et al., 2019; Rosano et al., 2019).

One of the main factors affecting metabolic burden is promoter strength. *lac-*derived promoters (P_*T7*_*_*lac*_*, P_trc_, and P_tac_) are all based on the negative regulation by LacI, and the expression is induced by lactose or non-metabolizable isopropyl β-D-1-thiogalactopyranoside (IPTG), used as analogous molecule of lactose, while pBAD plasmids carry a BAD promoter (P_BAD_) positively induced by L-arabinose (Müller-Hill et al., 1968; Silverstone et al., 1970; Terpe, 2006; Brautaset et al., 2009). P_trc_ and P_tac_ promoters are considered as strong promoters and are well characterized. Genes regulated by P_*T7*_ promoter are transcripted by the bacteriophage t7 RNA polymerase present in some *E. coli* strains, such as BL21 (DE3) (Phue et al., 2008). This polymerase is five times faster than *E. coli* RNA polymerase, so P_*T7*_*_*lac*_* is considered a very strong promoter (William Studier et al., 1990; Mertens et al., 1995). Although the induction system is different from *lac*, the P_BAD_ promoter is considered a medium-strong promoter, with a lower force of expression than P_tac_, P_trc_, and P_*T7*_*_*lac*_* (Guzman et al., 1995).

Replication origin (ori) of the replicon is the main component influencing the copy number of an expression vector and therefore in cell metabolic burden (Smolke and Keasling, 2002; Wang et al., 2009). There are many different origins including prokaryotic, eukaryotic, viral, and others unidentified (Wang et al., 2009). Specifically for enterobacteria, there are large differences in the number of copies related to the different origins (Jahn et al., 2016). For example, pMB1 (also known as pBR322) is related to medium copy number plasmids (15–20 copies/cell), some pMB1 derivatives have high copy number plasmids (500–700 copies/cell), while p15A is related to low PCNs (10 copies/cell) (Lin-Chao et al., 1992).

In this study, expression of the Kringle recombinant yellow fluorescent protein (KrYFP) has been evaluated in *E. coli* BL21 (DE3) using different expression systems. Thus, P_*T7*_*_*lac*_*, P_trc_, P_tac_, and P_BAD_ were selected as promoters to control YFP expression. Differences between these promoters have been studied in many works, although in most of them, the expression study has been carried out in different vector backbones (Lee and Keasling, 2005; Choi et al., 2010; Wu et al., 2010; Balzer et al., 2013). In the present work, selected promoters were cloned into a pet backbone with two different replication origins (high and low copy number). Induction was studied with two different carbon sources: glucose and glycerol. Moreover, protein expression system was tested in an *E. coli* BL21 Δ*ackA* strain, which shows higher recombinant protein production with respect to the BL21 wild-type strain due to the low acetate produced and excreted to the extracellular medium (Kim and Cha, 2003; Kim et al., 2015; Lozano Terol et al., 2019). Hence, we also expect to observe an increase in YFP expression.

Together, the results try to shed light on the process of expression vector and strain selection to optimize a recombinant protein production process, essential to achieve a successful yield. Moreover, results contributes to increase the metabolic burden knowledge to obtain synthetic biology models that allow predicting the behavior of the host cell and develop robust biofactory cells (Wu et al., 2016; Mühlmann et al., 2018).

## Materials and Methods

### Expression Vector Constructions

All primers and strains used in this study are listed in Table 1, and plasmids constructed and employed as templates are listed in Table 2. To construct expression vectors, the pSF-pA-PromMCS-KrYFP (pSF-pMB1′-YFP) plasmid was employed as a template. In order to replace the pMB1-derived ori (denoted as pMB1′ in this study), the pSF-pMB1′-YFP vector was amplified, except the original pMB1′ region, by using the pair of primers pSFYFP *Swa*I Fwd and pSFYFP *Pac*I Rev. The p15A ori was amplified from the pZ8-pTac plasmid employing p15A *Pac*I Fwd and p15A *Swa*I Rev primers. Both amplification products were digested with *Pac*I and *Swa*I restriction enzymes and ligated to obtain the plasmid pSF-p15A-YFP with the replication origin p15A. Construction of vectors were carried out with these two backbones, pSF-pMB1′-YFP and pSF-p15A-YFP, by inserting the promoter with regulator section in the multicloning site region of the plasmid. The P_*T7*_ promoter region and *lac* regulatory operator (*lacI*^*q*^ promoter, *lacO*, and *lacI* gene) were amplified from pet28a-MBP employing PT7 *Sal*I Fwd and PT7 *Hin*dIII Rev primers, amplified region and vectors were digested with *Sal*I and *Hin*dIII restriction enzymes, and ligated to obtain pSF-pMB1′-T7-YFP and pSF-p15A-T7-YFP vectors. Sequence coding P_trc_ promoter and *lac* regulatory operator was amplified from pTrcECT using PTrc *Sal*I Fwd and PTrc *Eco*RI Rev; both plasmids and PCR product were digested with *Sal*I and *Eco*RI restriction enzymes and were ligated to generate pSF-pMB1′-trc-YFP and pSF-p15A-trc-YFP vectors. Construction of pSF-pMB1′-tac-YFP and pSF-p15A-tac-YFP was carried out by P_tac_ promoter and regulatory *lac* operator from pZ8-pTac PCR using PTac *Sal*I Fwd and PTac *Hin*dIII Rev primers. Amplified sequence and backbone vectors were digested with *Sal*I and *Hin*dIII restriction enzymes and ligated to generate complete plasmids. The P_BAD_ promoter and *araC* regulatory gene were amplified from pBAD24 vector employing PBAD *Spe*I Fwd and PBAD *Eco*RI Rev. The PCR product was digested and inserted in both digested plasmids through *Spe*I and *Eco*RI restriction enzyme sites to generate pSF-pMB1′-BAD-YFP and pSF-p15A-BAD-YFP plasmids.

**TABLE 1:** Primers and strains used in this study.

**Primer**	**Sequence 5′→ 3′**	
PT7 *Sal*I Fwd	GGTGGT**GTCGAC**TCACTGCCCGCTTTCCAGT	
PT7 *Hin*dIII Rev	GGTGGT**AAGCTT**AGAGGGGAATTGTTATCCGC	
PTrc *Sal*I Fwd	GTTGTT**GTCGAC**GACACCATCGAATGGTGCAA	
PTrc *Eco*RI Rev	GTTGTT**GAATTC**TTGTTATCCGCTCACAATTCC	
PTac *SalI*Fwd	GTTGTT**GTCGAC**GACACCATCGAATGGTGCAA	
PTac *Hin*dIII Rev	GTTGTT**AAGCTT**CCGGGAATTCTGTTTCCTGT	
PBAD *Spe*I Fwd	GTTGTT**ACTAGT**TTATGACAACTTGACGGCTAC	
PBAD *Eco*RI Rev	GTTGTT**GAATTC**AAAAAAACGGGTATGGAGAAACAG	
p15A *Pac*I Fwd	GGTGGT**TTAATTAA**GGAAGATGCCAGGAAGATACT	
p15A *Swa*I Rev	GGTGGT**ATTTAAAT**TTTTCGTTCCACTGAGCGTCA	
pSFYFP *Swa*I Fwd	GGTGGT**ATTTAAAT**TTCCGAACTCTCCAAGGCC	
pSFYFP *Pac*I Rev	GGTGGT**TTAATTAA**GTTTCGATAGCCCAAGGTAACCAA	

**Strain**	**Description**	**Source**

Top10F′	F′[*lacI*^*q*^ Tn10(tet^R^)] *mcrA* Δ(*mrr-hsdRMS-mcrBC*) φ80*lac*ZΔM15 Δ*lacX74 deoR nupG recA1 araD139*Δ(*ara-leu*)7697 *galU galK rpsL*(StrR) *endA1* λ^–^	Invitrogen
BL21 (DE3)	F^–^ *ompT gal dcm lon hsdSB*(*rB^–^ mB^–^*) λ(DE3)	Promega
BL21 (DE3) Δ*ackA*	F^–^ *ompT gal dcm lon hsdSB*(*rB^–^ mB^–^*) λ(DE3) *ackA*:Kan^R^	Lozano Terol et al., 2019

*Restriction nuclease sites are resalted and are in bold.*

**TABLE 2:** Plasmids used and constructed in this study.

**Plasmid**	**Description**	**Source**
pet28a-MBP	pMB1 *ori*, *lacI* P_*T7*_ promoter, Kan^R^, Maltose Binding Protein (MBP) Phusion tag	Lab deposit
pTrcECT	pMB1 *ori*, *lacI*, P_trc_ promoter, Amp^R^. *ectABC* (Ectoine biosynthetic operon from *Halomonas elongate*) expression	pTrcECT was a gift from Xixian Xie Ning et al., 2016
pZ8-pTac	p15a *ori*, *lacI*, P_tac_ promoter, Kan^R^	pZ8-Ptac was a gift from Timothy Lu Cleto et al., 2016
pBAD24	pMB1 *ori*, L-arabinose P_BAD_ promoter, Amp^R^.	Lab deposit
pSF-pA-PromMCS-KrYFP (pSF-pMB1′-YFP)	pMB1′ *ori*, promotorless, Amp^R^. Kringle YFP (Yellow Fluorescence Protein) expression	Oxgene
pSF-p15A-YFP	p15A *ori*, promotorless, Amp^R^. Kringle YFP (Yellow Fluorescence Protein) expression	This study
pSF-pMB1′-t7-YFP	pMB1′ *ori*, *lacI* P_*T7*_ promoter, Amp^R^. Kringle YFP (Yellow Fluorescence Protein) expression	This study
pSF-pMB1′-trc-YFP	pMB1′ *ori*, *lacI*, P_trc_ promoter, Amp^R^. Kringle YFP (Yellow Fluorescence Protein) expression	This study
pSF-pMB1′-tac-YFP	pMB1′ *ori*, *lacI*, P_tac_ promoter, Amp^R^. Kringle YFP (Yellow Fluorescence Protein) expression	This study
pSF-pMB1′-BAD-YFP	pMB1′ *ori*, L-arabinose P_BAD_ promoter, Amp^R^. Kringle YFP (Yellow Fluorescence Protein) expression	This study
pSF-p15A-T7-YFP	p15A *ori*, *lacI* P_*T7*_ promoter, Amp^R^. Kringle YFP (Yellow Fluorescence Protein) expression	This study
pSF-p15A-trc-YFP	p15A *ori*, *lacI*, P_trc_ promoter, Amp^R^. Kringle YFP (Yellow Fluorescence Protein) expression	This study
pSF-p15A-tac-YFP	p15A *ori*, *lacI*, P_tac_ promoter, Amp^R^. Kringle YFP (Yellow Fluorescence Protein) expression	This study
pSF-p15A-BAD-YFP	p15A *ori*, L-arabinose P_BAD_ promoter, Amp^R^. Kringle YFP (Yellow Fluorescence Protein) expression	This study

### Growth and Expression Analysis

*Escherichia coli* BL21 (DE3) wild type or *ackA*-deficient strains were made competent by the rubidium chloride method (Hanahan, 1983). Chemically competent cells were transformed by heat shock at 42°C with the constructed pSF-pMB1′-YFP or pSF-p15A-YFP vectors. Transformed cells were grown in a Synergy H1 Hybrid Multi-Mode Reader to simultaneously measure growth at 600 nm (optical density OD_600_) and fluorescence, at 520 nm excitation and 542 nm emission. Then, 96-well sterilized plates were filled with 200 μl of complex TB7 medium with glucose (20 mM) or glycerol (40 mM) as carbon sources. To prevent evaporation and permit aeration, 96-well multiplates were covered with an adhesive gas-permeable sheet (Sigma Aldrich). Cultures were inoculated with precultures to an initial OD_600_ of 0.05 U and induced at 0.5 U with 0–2 mM IPTG or L-arabinose. TB7 composition was 10 g/L tryptone buffered at pH 7.0 with 100 mM K_2_HPO_4_. Cultures were grown in triplicate with double orbital shaking at 37°C for 48 h. The specific growth rate was determined as previously described (Lozano Terol et al., 2019).

### YFP Concentration Determination

In order to quantify YFP produced, cultures at stationary growth step were harvested at 4,000 × *g* for 15 min at 4°C. Cells were disrupted by sonication for 2 min (40 s each pulse) using a Vibra Cell sonicator (Sonicator Sonics & Materials, Newton, United Kingdom). Lysates were analyzed by electrophoresis SDS-PAGE with 10% acrylamide gels using Mini-PROTEAN Tetra Cell (Biorad, California, CA, United States) followed by Coomassie staining (Fisher Scientific, Madrid, Spain). Standard curve was constructed to calculate YFP concentration by densitometric analysis using ImageJ Gel Analyzer software (Rueden et al., 2017).

### Recombinant Protein Solubility Study

To evaluate the solubility of the overexpressed YFP protein, chemically competent cells harboring constructed pSF-pMB1′-YFP or pSF-p15A-YFP vectors were grown overnight in batch mode at 37°C with orbital shaking (250 rpm). Culture medium and inductor concentration were selected in expression analysis. Pellets were harvested by centrifugation (20 min; 4,500 × *g*) and resuspended in native buffer (50 mM K_2_HPO_4_, 500 mM NaCl, pH 8). Cells were disrupted by sonication for 2 min (40 s each pulse) using a Vibra Cell sonicator (Sonicator Sonics & Materials, Newton, United Kingdom). The lysates were clarified by centrifugation at 14,000 × *g* for 30 min at 4°C to obtain supernatants (soluble protein extracts). Pellets were resuspended again with denatured buffer (50 mM K_2_HPO_4_, 500 mM NaCl, urea 6 M, pH 8) and incubated under shaking for 30 min. Finally, cells were centrifugated at 14,000 × *g* for 30 min at 4°C to isolate supernatants (insoluble protein extracts). To analyze YFP solubility, electrophoresis SDS-PAGE with 10% acrylamide gels was carried out. Protein gels were run under denaturing conditions using Mini-PROTEAN Tetra Cell (Biorad, California, CA, United States) followed by Coomassie staining (Fisher Scientific, Madrid, Spain). ImageJ Gel Analyzer software was used for densitometric quantification (Rueden et al., 2017).

## Results and Discussion

### Construction of Two Different Replication Sets of Expression Vectors

**FIGURE 1:**
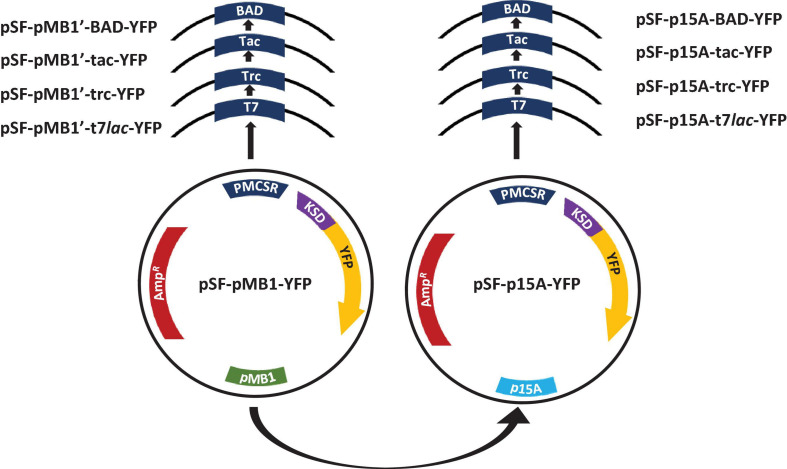
Scheme of expression vectors constructed in this study. PMCSR, promoter multicloning site region; KSD, Kozak Shine-Dalgarno; YFP, yellow fluorescent protein; Amp^R^, ampicillin resistance.

To evaluate the influence of replication origin on expression of recombinant proteins in *E. coli* BL21, p15A (∼10 copies/cell) and a high copy number derived from pMB1 ori (500–700 copies/cell), denoted as pMB1′, were selected (Selzer et al., 1983; Lin-Chao et al., 1992). To avoid influence by other vector components in expression, a common backbone was chosen for all constructions with the same ribosomal binding site sequence (Shine Delgarno sequence), the vector pSF-pMB1’-YFP (pMB1’ origin) (Table 1). The pSF-p15A-YFP was built from this, which was exactly the same as the previous one, except for the origin (p15A). One of the most important aspects to consider when designing a recombinant protein production process is the choice of the promoter system. Together with the replication system, it will be decisive for the level of expressed recombinant protein. We selected the prokaryotic P_*T7*_*_*lac*_*, P_trc_, P_tac_, and P_BAD_ promoters. The first three are based on the negative regulation by LacI^Q^ and the last induced by L-arabinose. A scheme of the plasmids constructed for this study is shown in Figure 1. The resulting plasmids were named pSF-pMB1′-T7*lac*-YFP (6,131 bp), pSF-pMB1′-trc-YFP (6,059 bp), pSF-pMB1′-tac-YFP (5,969 bp), pSF-pMB1′-BAD-YFP (5,815 BP), pSF-p15A-T7lac-YFP (5,749 bp), pSF-p15A-trc-YFP (5,677 bp), pSF-P15A-tac-YFP (5,587 bp), and pSF-p15A-BAD-YFP (5,433 bp).

### Yellow Fluorescent Protein Expression Under Different Induction Conditions

**FIGURE 2:**
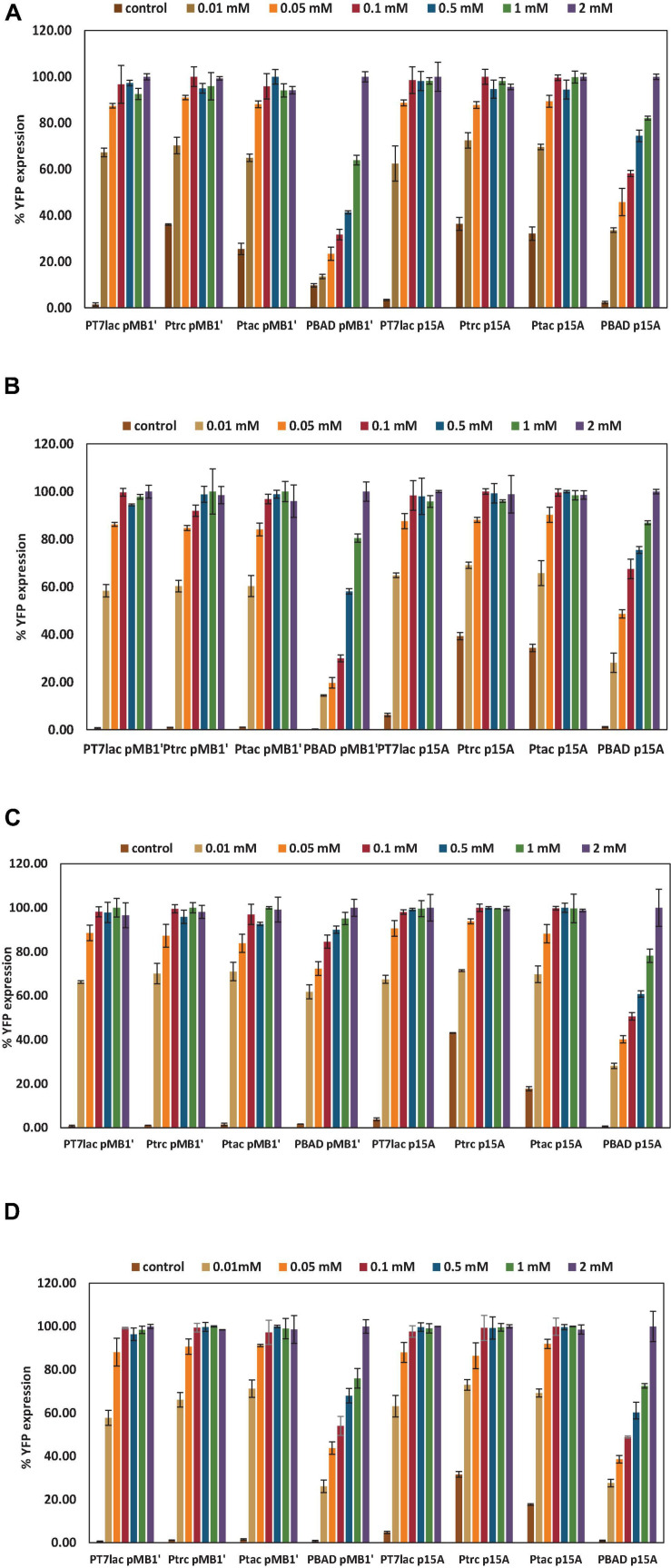
Percentage of yellow fluorescent protein (YFP) expression with respect to maximal expression for each plasmid under different inductor conditions. The YFP expressions selected were the highest achieved at stationary growth phase. **(A)** *E. coli* wt growing with glucose as carbon source. **(B)** *E. coli* wt growing with glycerol as carbon source. **(C)** *E. coli* Δ*ackA* growing with glucose as carbon source. **(D)** *E. coli* Δ*ackA* growing with glycerol as carbon source.

To know the behavior of an expression system under different inductor concentrations is essential to optimize a protein expression process. Here, to study how the constructed plasmids expressed the recombinant YFP, different induction conditions were evaluated. Thus, IPTG and L-arabinose 0 (control), 0.01, 0.05, 0.1, 0.5, 1, and 2 mM were tested, and maximal expressions observed for each vector were normalized to 100% (Figure 2). As shown in Figure 2, all *lac*-based systems showed the highest YFP expression from 0.1 mM IPTG, while for the P_BAD_ promoter, it was necessary to have a 2 mM L-arabinose concentration. Moreover, induction profiles were similar in glucose and glycerol cultures and for wt or *ackA*-deficient mutant of *E. coli*. Thus, 0.1 mM was selected as the optimal IPTG concentration for *lac*-based vectors and 2 mM for vectors with P_BAD_. The difference in optimal concentration of inducers is probably a consequence of all-or-none expression. The P_BAD_ and *lac* promoters give rise to a gene expression known as all-or-none when induced with natural lactose or L-arabinose, respectively. This phenomenon refers to the fact that, at sub-saturated concentrations of inducer, a homogeneous level of induction is not obtained, but rather cultures in which there is a percentage of cells totally induced and another that is not induced. The gratuitous inducer IPTG is a non-metabolizable lactose analog that can freely cross the cell wall and membrane. In this sense, using IPTG as inducer, homogeneous cultures are achieved at different concentrations, which allows us to optimize the expression in a tighter way, and to use lower concentrations of inducer (Khlebnikov and Keasling, 2002). However, at the moment, no analog to L-arabinose is known that allows to eliminate this phenomenon (Afroz et al., 2014).

**FIGURE 3:**
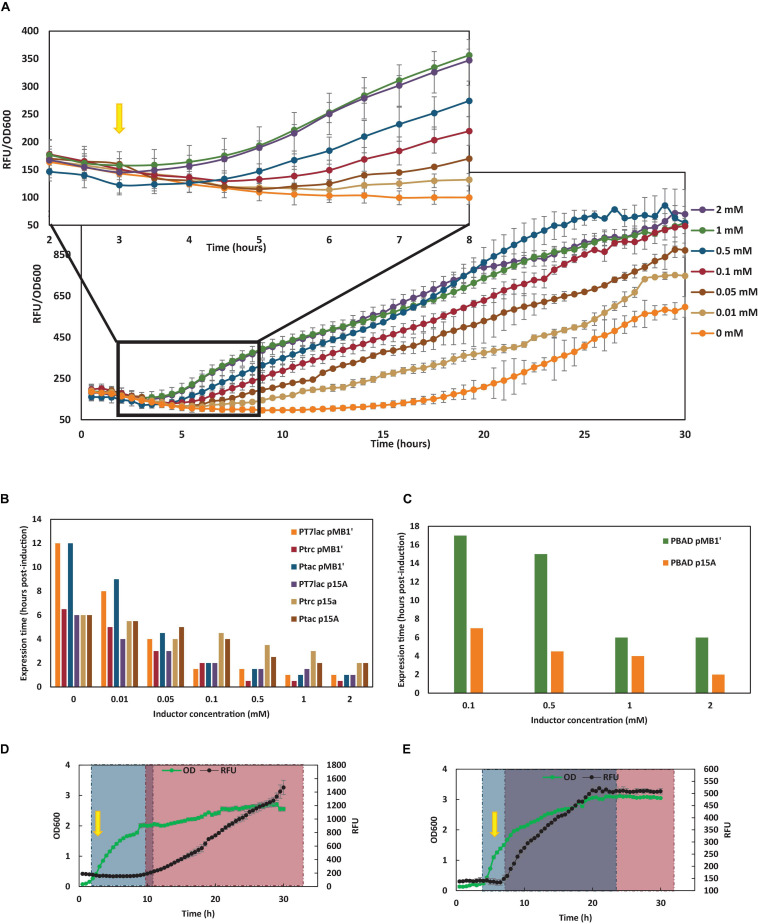
Dependence of inductor on YFP expression time. **(A)** YFP expression of *E. coli* wt carrying the pSF-pMB1’-tac-YFP and growing with glucose as carbon source at different IPTG concentrations. Yellow arrow shows induction time with 0.1 mM IPTG. **(B)** YFP expression time (in hours after 0.1 mM IPTG addition) of *E. coli* wt carrying the different *lac* plasmids constructed growing in TB7 supplemented with glucose as carbon source at different IPTG concentrations. **(C)** YFP expression time (in hours after 2 mM L-arabinose addition) of *E. coli* wt carrying the P_BAD_ plasmids constructed growing in TB7 supplemented with glucose as carbon source at different L-arabinose concentrations. **(D)** *E. coli* wt carrying pSF-pMB1′-BAD-YFP growing with glucose as carbon source. **(E)** *E. coli* wt carrying pSF-pMB1′-BAD-YFP growing with glycerol as carbon source. Green dots show grown at 600 nm, while black dots show YFP fluorescence in arbitrary units (RFU). Gray square corresponds with culture growth, and pink square indicates YFP expression. Purple square indicates hours when culture is growing and expressing YFP simultaneously. Yellow arrow shows 2 mM L-arabinose induction time.

As shown in Figure 2, in most conditions assayed, a basal expression without inducer was observed at long culture times (see also control expression in Figure 3A). This basal expression was negligible in P_BAD_ promoter-based vector because AraC represses translation and L-arabinose is absolutely needed for induction (Schleif, 2010). The highest basal expression was observed for P_trc_ and P_tac_ promoter vectors. *lac* promoters are known to have leaky transcription, that is, transcription occurs when the inducer is absent (Rosano and Ceccarelli, 2014). This fact was observed in cultures transformed with P_trc_ and P_tac_ p15A vectors, in spite of carrying the LacQ improved version (Penumetcha et al., 2010). However, P_*T7*_*_*lac*_* showed a low basal expression, in spite of being a *lac* promoter, probably due to double repression (both, P_*T7*_*_*lac*_* promoter and phage T7 RNA polymerase). Basal expression observed in P_trc_ and P_tac_ promoter vectors could be a drawback when a toxic protein for cell is overexpressed.

### Inducer Concentration Influence on Yellow Fluorescent Protein Expression Time

In order to deepen the influence of inducer concentration on YFP production, expression rates were calculated for all the cultures (data not shown), but these rates were not affected by inductor concentration. However, the inducer concentration was fundamentally reflected in the decrease in the time of YFP expression. Thus, in all cases, as the inducer concentration increased, the expression of the recombinant protein was observed earlier. In Figure 3A, the YFP expression of *E. coli* wt carrying the pSF-pMB1′-tac-YFP and growing with glucose as carbon source at different IPTG concentrations is shown. Basal expression was observed in control culture (IPTG 0 mM) at long culture times. However, when IPTG was present, expression was observed earlier, although from IPTG, 0.1 mM differences in the time of expression were almost negligible. The same expression profile was observed for all cultures carrying *lac* vectors. In Figure 3B, expression time (in hours after the inducer addition) is shown with respect to the concentration of inducer added for *E. coli* wt transformed with the six constructed *lac* vectors growing in TB7 supplemented with glucose. As can be seen, in the absence of inductor, basal expression started at 6–17 h post-induction, due to insufficient LacI expression. However, at 0.1 mM IPTG, YFP expression was observed at 1–4 h after induction. Cultures carrying P_BAD_ vectors did not show expression until L-arabinose was at 0.1 mM, due to the all-or-none phenomenon (Figure 3C). Moreover, expression times were much longer in P_BAD_ than in *lac* vectors when glucose was the sole carbon source due to catabolite repression (Figures 3D,E). Hence, P_BAD_ promoter is regulated by this phenomenon, in addition to L-arabinose induction. Due to the non-PTS nature of glycerol, catabolite repression is not observed when cultures are supplemented with glycerol as carbon source (Figure 3E).

### Effects of the Different Expression Systems and Conditions on the Yellow Fluorescent Protein Expression

**FIGURE 4:**
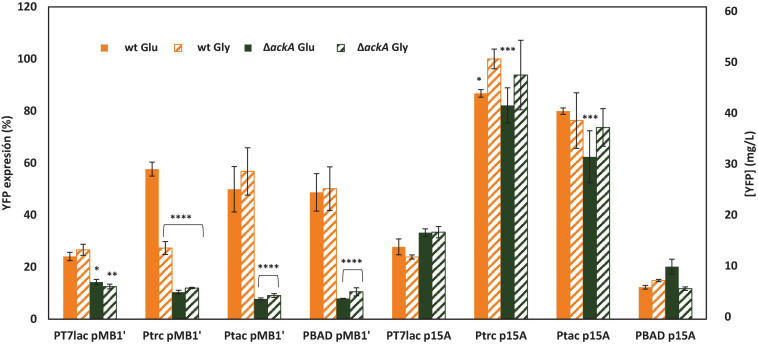
YFP expression of *E. coli* wt or deficient *ackA* mutant growing with glucose or glycerol as carbon sources carrying different expression system vectors. Expression is shown as a percentage with respect to expression of *E. coli* wt transformed with the plasmid pSF-p15A-trc-YFP growing with glycerol as the 100%. Secondary y-axis shows YFP concentration (mg/L). Expression of *E. coli* wt is shown in orange and *E. coli*Δ*ackA* in green. Glucose cultures are in solid bars, while glycerol supplemented cultures are shown in striped bars.

In order to compare the expression of the recombinant protein YFP under the selected induction conditions (0.1 mM IPTG and 2 mM L-arabinose) with the different constructed vectors, triplicates of each combination were carried out in the same multiwell plate. In this way, it was possible to compare the expression of YFP in *E. coli* wt and Δ*ackA* mutant growing with glucose or glycerol and transformed with each plasmid. Figure 4 shows the expression observed at stationary growth phase in each culture. The expression is shown in percentage with respect to the maximum expression reached (*E. col*i wt growing with glycerol as carbon source transformed with the plasmid pSF-p15A-trc-YFP). Moreover, YFP expressed was quantified, and the concentration (mg of protein/L of culture) is also shown. Statistical testing involving two-way ANOVA was carried out with Graphpad Prism 7.0 in order to evaluate statistically significant differences between maximal expression condition observed in each plasmid with respect to the other conditions [*p*-value < 0.0001 (^****^), <0.001 (^∗∗∗^), <0.01 (^∗∗^), and <0.05(^∗^)].

Results showed that the highest expression was achieved with the vectors with the lowest number of copies, which contained the p15A origin. High copy number vectors have been previously associated to a lower protein production than medium and low copy plasmids (Jones et al., 2000; Silva et al., 2012). On the contrary, P_BAD_ showed higher YFP expression with high copy plasmid, probably due to the weaker strength of BAD promoter with respect to *lac*. Thus, the combination of a high copy number origin of replication and a strong promoter caused a metabolic mismatch, which triggered a decrease in YFP production. This metabolic burden has been, and continues to be, widely studied, since it implies a loss of productivity (Wu et al., 2016). Thus, not only the production of recombinant proteins but also the presence of the plasmid in the host cell has several metabolic and physiological consequences such as alterations in growth rate or differential expression of essential metabolic enzymes (Silva et al., 2012).

In order to increase the knowledge between YFP expression and metabolic burden, growth rates were calculated for those cultures with the highest YFP expression observed for each promoter. Moreover, growth rate of wt and *ackA* mutant without any plasmid and containing the expression vector without any promoter were also calculated. Growth curves of empty strains and containing promoterless vectors are shown in Supplementary Figure 1, and growth rates are shown in Table 3. Empty wt and *ackA-*deficient strains showed the highest rates. Strains containing vector without any promoter showed a great decrease in growth rate, revealing a metabolic mismatch due to replication and maintenance of the additional plasmid DNA in the cell. Comparing strains containing pMB1′ with p15A vectors, high copy number plasmids caused a greater decrease in growth rate. Knowledge about why this metabolic imbalance occurs is still insufficient, certain studies point to a collapse in the cellular translation machinery in the face of an excess of extrinsic mRNA from the heterologous gene/genes, which is in concordance with growth rates observed in this study (Mairhofer et al., 2013; Tan et al., 2020). Moreover, decrease in growth rate also showed a dependence on strength promoter although lower than replication origin. This result highlights that metabolic burden is mainly due to transcription and, to a lesser extent, to recombinant protein translation, which has been recently discussed (Li and Rinas, 2020).

**TABLE 3:** Growth rates calculated for empty strains and containing promoterless vectors growing with glucose or glycerol as carbon sources.

	***E. coli* strain**	**Carbon source**	**Expression vector**	**Growth rate**
wt Glu	BL21	Glucose	No vector (empty strain)	0.99 ± 0.04
wt Gly	BL21	Glycerol	No vector (empty strain)	0.72 ± 0.03
wt Glu pMB1′	BL21	Glucose	No promoter-YFP pMB1′	0.54 ± 0.05
wt Gly pMB1′	BL21	Glycerol	No promoter-YFP pMB1′	0.43 ± 0.03
wt Glu p15A	BL21	Glucose	No promoter-YFP p15A	0.63 ± 0.03
wt Gly p15A	BL21	Glycerol	No promoter-YFP p15A	0.53 ± 0.1
ΔackA Glu	BL21 Δ*ackA*	Glucose	No vector (empty strain)	0.56 ± 0.05
ΔackA Gly	BL21 Δ*ackA*	Glycerol	No vector (empty strain)	0.50 ± 0.07
ΔackA Glu pMB1′	BL21 Δ*ackA*	Glucose	No promoter-YFP pMB1′	0.19 ± 0.02
ΔackA Gly pMB1′	BL21 Δ*ackA*	Glycerol	No promoter-YFP pMB1′	0.18 ± 0.02
ΔackA Glu p15A	BL21 Δ*ackA*	Glucose	No promoter-YFP p15A	0.42 ± 0.03
ΔackA Gly p15A	BL21Δ*ackA*	Glycerol	No promoter-YFP p15A	0.30 ± 0.03
P_*T7lac*_ pMB1′	BL21	Glucose	P_*T7lac*_-YFP pMB1′	0.31 ± 0.03
P_trc_ pMB1′	BL21	Glucose	P_trc_-YFP pMB1′	0.21 ± 0.01
P_tac_ pMB1′	BL21	Glycerol	P_tac_-YFP pMB1′	0.22 ± 0.01
P_BAD_ pMB1′	BL21	Glycerol	P_BAD_-YFP pMB1′	0.17 ± 0.02
P_*T7lac*_ p15A	BL21 Δ*ackA*	Glycerol	P_*T7lac*_-YFP p15A	0.24 ± 0.03
P_trc_ p15A	BL21	Glycerol	P_trc_-YFP p15A	0.5 ± 0.05
P_tac_ p15A	BL21	Glucose	P_tac_-YFP p15A	0.65 ± 0.05
P_BAD_ p15A	BL21 Δ*ackA*	Glycerol	P_BAD_-YFP p15A	0.28 ± 0.04

*Growth rates of cultures with the highest YFP expression observed for each promoter were also calculated. *E. coli* strain and carbon source of each culture are shown.*

Glycerol has become a potential alternative to glucose, the traditional carbon source, due to its lower cost as a subproduct of biodiesel production (Clomburg and Gonzalez, 2013). Results observed in this study show a similar expression for cultures growing with glucose or glycerol as carbon source, so glycerol could be a better alternative without a decrease in the final yield. Regarding the *ackA*-deficient mutant, expression with high copy vectors was much lower than that observed for the wt strain. However, when low copy plasmids were employed, Δ*ackA* showed a similar expression to wt. The low expression measured with pMB1′ was probably due to an increase in the metabolic burden caused by *ackA* gene depletion, which agrees with the growth rates. Thus, *ackA-*deficient mutant showed approximately half of wt growth rate. Previous studies have observed an increase in recombinant protein expression when *E. coli* BL21 *ackA* knockout strain was used as host. Thus, *ackA* depletion avoids acetate overflow and energy waste associated to it. However, none of these studies used a plasmid with such a high copy number as pMB1′ (Kim and Cha, 2003; Kim et al., 2015; Lozano Terol et al., 2019). To our knowledge, this is the first expression study carried out in *E. coli* BL21 Δ*ackA* with a high copy number plasmid and a strong promoter.

**FIGURE 5:**
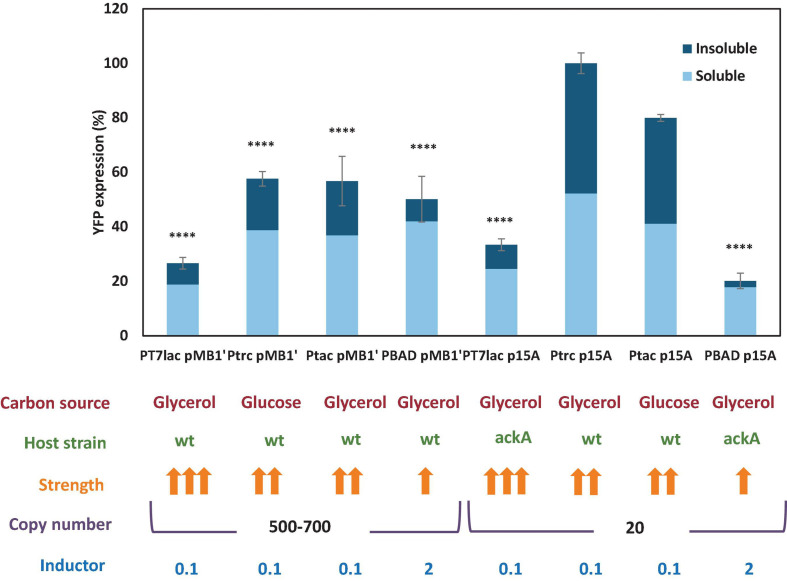
Soluble and insoluble percentage of YFP measured for each expression vector. The fluorescence is indicated as a percentage, with 100% as observed with the pSF-p15A-trc-YFP vector. Carbon source, host strain, strength of the promoter, inductor concentration, and vector copies are shown under the graph. Statistical analysis by one way ANOVA was carried out with Graphpad Prism 7.0 [*p* value < 0.0001 (^****^), <0.001 (^∗∗∗^), <0.01 (^∗∗^), and <0.05(^∗^)].

To compare all conditions, statistical testing involving two-way ANOVA of the maximal YFP expression achieved with each vector was carried out (Figure 5). Figure 5 shows significant differences of maximal expression compared with YFP expression with pSF-p15A-trc-YFP, the highest observed. Quantitative analysis revealed a YFP maximal concentration of 53.09 mg/L, which agrees with other studies focused on GFP production in *E. coli* (Chew and Tan, 2012; Fragoso-Jiménez et al., 2019). All expressions, except the ones corresponding to pSF-p15A-tac-YFP were significantly lower. Thus, for low copy vectors, P_trc_ promoter achieved threefold higher expression than P_*T7*_*_*lac*_* and 5.5-fold than the P_BAD_. It is interesting to highlight how the joint influence of the promoter strength and the number of copies associated with each of the two replication origins used is observed. Thus, for P_*T7*_*_*lac*_*, the strongest promoter of the four studied, the expression of YFP is lower than for P_trc_ and P_tac_, both in the low and high copy number vector. This low expression might be due to the metabolic stress caused by excess transcripts or to an insufficient expression of RNA T7 polymerase (Vethanayagan and Flower, 2005; Mairhofer et al., 2013). These results indicate the need to find an adequate balance between these two factors, which will need to be optimized for each production process.

### Study of Recombinant Yellow Fluorescent Protein Protein Solubility

One of the greatest drawbacks to obtain high recombinant protein yields with *E. coli* is the formation of inclusion bodies and protein precipitation, due to high expression, incorrect folding, aggregation, or low chaperone activity (Gopal and Kumar, 2013). Recombinant protein precipitation involves, in addition to a decrease in the yield of the production process, an alteration in the gene transcription of the strain (Baig et al., 2014). Hence, to optimize a large-scale recombinant protein production process, it is essential to know what proportion of the protein forms precipitates and what fraction remains in soluble form, and therefore functional for most subsequent applications. In order to know the percentage of soluble and insoluble YFP protein, batch cultures were carried out with *E. coli* growing under the conditions corresponding to the maximum expression observed for each vector (Figure 5). When cultures reached the stationary phase, soluble/insoluble fractions were analyzed by electrophoresis SDS-PAGE and subsequent densitometric analysis. Electrophoresis gels are shown in Supplementary Figure 2. Figure 5 results showed that insoluble YFP protein was present in all cultures. However, cultures where YFP was expressed under P_BAD_ promoter control showed lower insoluble fraction. Moreover, cultures containing pSF-p15A-trc-YFP and pSF-p15A-tac-YFP vectors, of which YFP expression was the highest, showed a similar percentage of soluble and insoluble protein. This difference observed in the amount of protein precipitated according to the expression plasmid used is very relevant, since it is useless to achieve a high expression if most of the protein is precipitated together with the cell pellet. Therefore, this aspect should be studied previously when selecting an expression system, especially if we are faced with a protein with limited solubility.

## Conclusion

Production of recombinant proteins has become an essential process to obtain drugs and other metabolites with high industrial interest. *E. coli*, as a prokaryotic model, is often the host of choice to produce proteins or other metabolites, especially when these proteins do not require complex post-translational modifications (Rosano and Ceccarelli, 2014). One of the advantages of *E. coli* as a host is the wide variety of expression vectors available. These expression vectors have different components, such as promoters or origins of replication, which are essential to tune the expression of our proteins of interest.

Metabolic burden associated to expression of heterologous proteins in microbial hosts is known to be one of the main drawbacks to achieve high recombinant protein yields. As has been demonstrated in this study, the negative effects of this imbalance can be minimized by tuning heterologous gene expression through vector copy number-promoter strength balance. Therefore, studies on plasmid copy number combined with the type of replication origin and promoter characteristics give important information to improve synthetic biology in heterologous protein and metabolite production method application (Koma et al., 2018; Shariati et al., 2021). In conclusion, the results show the importance of the transcription system optimization according to the characteristics of each process to achieve a successful result.

## Data Availability Statement

The original contributions presented in the study are included in the article/Supplementary Material, further inquiries can be directed to the corresponding author/s.

## Author Contributions

GLT, JG-J, and TDP conceptualized the study. JG-J prepared and wrote the original draft. JG-J, GLT, RSM, AMV, MCD, and TDP wrote, reviewed, and edited the manuscript. TDP performed the supervision and administration, and acquired the funding. All authors have read and agreed to the published version of the manuscript.

## Conflict of Interest

The authors declare that the research was conducted in the absence of any commercial or financial relationships that could be construed as a potential conflict of interest.
