# Supplementary material for: Impact of the Expression System on Recombinant Protein Production in Escherichia coli BL21
Source: Front Microbiol. 2021 Jun 21;12:682001. doi: 10.3389/fmicb.2021.682001 (PMC8257044; doi:10.3389/fmicb.2021.682001)
Supplement: Supplementary file 1 [file Data_Sheet_1.PDF]

Supplementary Material

Impact of the expression system on recombinant protein production in *E. coli* BL21

Gema Lozano-Terol[+], Julia Gallego-Jara[+], Rosa A. Sola-Martínez, Adrián Martínez Vivancos, Manuel Cánovas Díaz and Teresa de Diego Puente[*]

Department of Biochemistry and Molecular Biology and Immunology (B), Faculty of Chemistry, University of Murcia, Campus of Espinardo, Regional Campus of International Excellence ''Campus Mare Nostrum'', P.O. Box 4021, Murcia E-30100, Spain.

[+]These authors contributed equally to this article

[*]Corresponding author (Email: tdp@um.es ; Tel.: +34868887397; Fax: +34968364148)

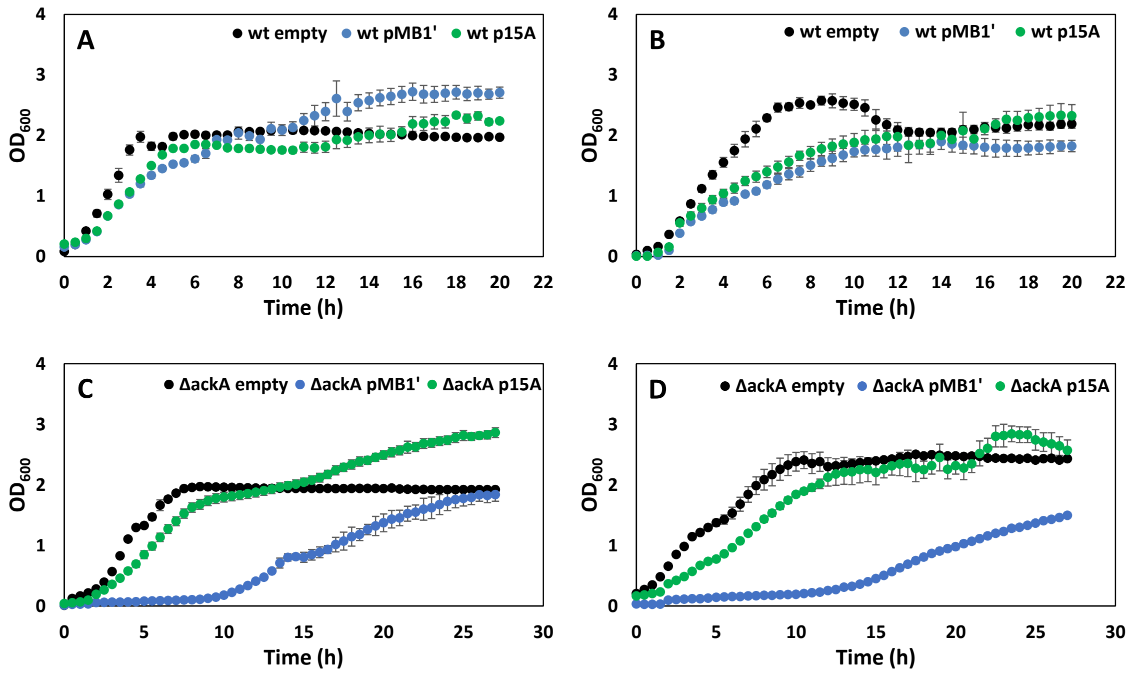

Figure S1. *E. coli* BL21 wt and Δ*ackA* strains without any plasmid (empty) and containing the high (pMB1') and low (p15A) copy number expression vectors without any promoter grown at OD_{600}. **A.** *E. coli* BL21 wt empty or containing vectors growing in TB7 supplemented with glucose. **B.** *E. coli* BL21 wt empty or containing vectors growing in TB7 supplemented with glycerol. **C.** *E. coli* BL21 Δ*ackA* empty or containing vectors growing in TB7 supplemented with glucose. **D.** *E. coli* BL21 Δ*ackA* empty or containing vectors growing in TB7 supplemented with glycerol.

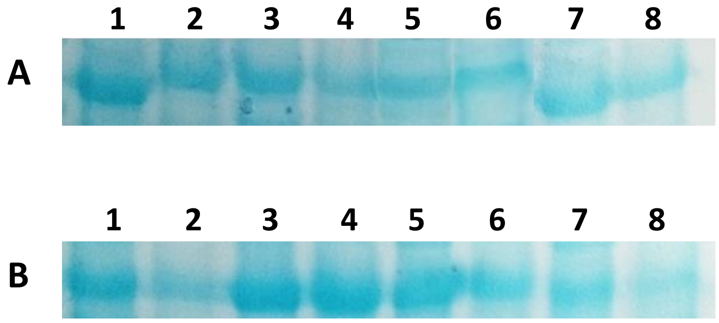

Figure S2. Electrophoresis SDS-PAGE gels of soluble/insoluble protein fractions corresponding to YFP. **A.** Fractions with soluble/insoluble YFP expressed with high copy number expression vectors (pSF-pMB1'-YFP). **B.** Fractions with soluble/insoluble YFP expressed with high copy number expression vectors (pSF-p15A-YFP). **Lane 1**, soluble YFP fraction expressed under $P_{T7/\mathit{lac}}$ control. **Lane 2**, insoluble YFP fraction expressed under $P_{T7/\mathit{lac}}$ control. **Lane 3**, soluble YFP fraction expressed under P_{trc} control. **Lane 4**, insoluble YFP fraction expressed under P_{trc} control. **Lane 5**, soluble YFP fraction expressed under P_{tac} control. **Lane 6**, insoluble YFP fraction expressed under P_{tac} control. **Lane 7**, soluble YFP fraction expressed under P_{BAD} control. **Lane 8**, insoluble YFP fraction expressed under P_{BAD} control.
